# Performance evaluation of a multinational data platform for critical care in Asia

**DOI:** 10.12688/wellcomeopenres.17122.2

**Published:** 2022-07-11

**Authors:** Collaboration for Research, Implementation and Training in Critical Care - Asia Investigators, Luigi Pisani, Thalha Rashan, Maryam Shamal, Aniruddha Ghose, Bharath Kumar Tirupakuzhi Vijayaraghavan, Swagata Tripathy, Diptesh Aryal, Madiha Hashmi, Basri Nor, Yen Lam Minh, Arjen M. Dondorp, Rashan Haniffa, Abi Beane

**Affiliations:** 1 Mahidol Oxford Tropical Research Unit, Bangkok, Thailand; 2 Doctors with Africa CUAMM, Padova, Italy; 3 NICS-MORU collaboration, Crit Care Asia Afghanistan team, Kabul, Afghanistan; 4 Department of Medicine, Chattogram Medical Centre, Chattogram, Bangladesh; 5 Indian Registry of IntenSive care, IRIS, Chennai, India; 6 Chennai Critical Care Consultants, Chennai, India; 7 Critical Care Medicine,, Apollo Hospitals, Chennai, India; 8 Anaesthesia and Intensive Care Medicine, All India Institute of Medical Sciences, Bhubaneswar, India; 9 Critical Care and Anesthesia, Nepal Mediciti Hospital, Lalitpur, Nepal; 10 Department of Critical Care, Ziauddin University, Karachi, Pakistan; 11 Department of Anaesthesiology and Intensive Care, Kulliyyah (School) of Medicine,, International Islamic University Malaysia (IIUM), Kuala Lumpur, Malaysia; 12 Oxford University Clinical Research Unit, Ho Chi Minh City, Vietnam

**Keywords:** case mix; critical care; high-quality clinical database; intensive care units; low and middle income country; ICU; registry; Asia.

## Abstract

**Background:** The value of medical registries strongly depends on the quality of the data collected. This must be objectively measured before large clinical databases can be promoted for observational research, quality improvement, and clinical trials. We aimed to evaluate the quality of a multinational intensive care unit (ICU) network of registries of critically ill patients established in seven Asian low- and middle-income countries (LMICs).

**Methods:** The Critical Care Asia federated registry platform enables ICUs to collect clinical, outcome and process data for aggregate and unit-level analysis. The evaluation used the standardised criteria of the Directory of Clinical Databases (DoCDat) and a framework for data quality assurance in medical registries. Six reviewers assessed structure, coverage, reliability and validity of the ICU registry data. Case mix and process measures on patient episodes from June to December 2020 were analysed.

**Results:** Data on 20,507 consecutive patient episodes from 97 ICUs in Afghanistan, Bangladesh, India, Malaysia, Nepal, Pakistan and Vietnam were included. The quality level achieved according to the ten prespecified DoCDat criteria was high (average score 3.4 out of 4) as was the structural and organizational performance -- comparable to ICU registries in high-income countries. Identified strengths were types of variables included, reliability of coding, data completeness and validation. Potential improvements included extension of national coverage, optimization of recruitment completeness validation in all centers and the use of interobserver reliability checks.

**Conclusions:** The Critical Care Asia platform evaluates well using standardised frameworks for data quality and equally to registries in resource-rich settings.

## Introduction

The availability of high quality data systems to inform delivery, evaluation and improvement of health care is recognised as a central tenet of high quality health systems ^1^. In critical care, where patient populations are heterogeneous, treatments complex and where the sequelae of care requires considerable human and financial resource, intensive care unit (ICU) registries have been instrumental in providing a mechanism for continuous, sustainable, wide scale data collection to enable service evaluation and facilitate national benchmarking of care quality. Until recently, these registries have been concentrated in high income countries, with the notable exceptions of networks in Brazil ^2^. and Sri Lanka ^3^ Absence of these systems in resource constrained countries severely hamper efforts to build accountability for healthcare quality.

The need to invest in systems which provide data to drive research and improvement has been highlighted by recent recommendations as part of a series of strategies to address the imbalance in quality of care that exists internationally ^1^. Recent growth in global internet connectivity and mobile technology has given opportunity for the digital health information system to be implemented and scaled in low and middle-income countries (LMICs).

The global coronavirus disease 2019 (COVID-19) pandemic has accelerated the role of registries in driving global research. For example, registries in Brazil, Australia, Europe, and in Asia have been instrumental as part of collaborations for pre-COVID-19 large scale multicentre studies ^4, 5^, observational research on COVID-19 ^6^ and more recently interventional research, as exemplified by the randomized, embedded, multi factorial adaptive platform for community acquired pneumonia (REMAP-CAP) operational through registries in the USA and in South Asia ^6^.

Whilst registries are increasingly being promoted for their role in enabling greater accountability of healthcare quality, and for their ability to facilitate multi centre clinical trials, the quality of data such systems provide requires rigorous evaluation ^7, 8^. To date, evaluation of existing vertical programme assessments for digital clinical and research registries, and for the World Health Organisation (WHO) endorsed district health information system platform ^9^, have focused predominantly on the ongoing challenges of missingness and inaccuracies in reporting ^10^. Few evaluations have extended to assess the timeliness, consistency, interoperability and accessibility of the data for external comparison ^11, 12^, despite these dimensions of data quality being essential for clinical research ^13^.

This study evaluates a network of seven federated registries operational in Asia which together use a single cloud-based platform as part of a collaboration for implementation and research in critical care. Critical Care Asia (CCA) is a collaborative programme of critical care research, training and quality improvement in Asia ^14^. The CCA currently connects 97 ICUs in seven countries to provide diverse high-quality data to generate evidence and feedback in near real time for service improvement and research, akin to the foundations of a learning health system ^15 ^. We sought to systematically evaluate the performance of CCA registries in Afghanistan, Bangladesh, India, Malaysia, Nepal, Pakistan and Vietnam using two pre published quality assurance frameworks ^16, 17^. We hypothesized that the quality of data arising from this federated network of registries would be high and comparable to the quality arising from ICU registries in high-resource settings.

## Methods

### Ethical considerations

This performance evaluation was classified as an audit and exempted from ethical review by the Oxford Tropical Research Ethics Committee (OxTREC) on June 16 ^th^, 2020. The evaluation was conducted on registry data collected between June and December 2020.

### Frameworks for assessment of performance

The Directory of Clinical Databases (DoCDat) framework was established to inform researchers and clinicians on currently functioning clinical databases and to provide an independent assessment of their scope and quality ^16^. Several high quality national registries in Australia, New Zealand and in the United Kingdom have used this same framework to evaluate data quality previously ^11, 12^. The framework ( Table 1) consists of 10 items; four relating to registry coverage and six relating to reliability and validity of the data. Each item is rated on a scale of 1 to 4, with level 1 representing the least rigorous methods and Level 4 representing the most rigorous. The instrument was shown to have good face and content validity and to have no floor or ceiling effects ^16^. A further framework to objectively assess registry quality especially in the development and implementation phase was published in 2002 and is also used in this evaluation ( Table 2) ^17^. This framework is divided into three main categories, and each category was applied to the central coordinating center and to the local sites. In case of disagreement between reviewers, final scoring was reached by consensus.

**Table 1.** Directory of Clinical Databases (DoCDat) scoring criteria.

Domain	Level 1	Level 2	Level 3	Level 4
**A. Extent to which the eligible** **population is representative** **of the country**	No evidence or unlikely to be representative	Some evidence eligible population is representative	Good evidence eligible population is representative	Total population of country included
**B. Completeness of** **recruitment of eligible** **population. State when** **and how completeness was** **determined**	Few (<80%) or unknown	Some (80–89%)	Most (90–97%)	All or almost all (>97%)
**C. Variables included in the** **database**	• identifier • admin info • condition or intervention	• identifier • admin info • condition **or** intervention • short term **or** long term outcome	• identifier • admin info • condition • intervention • short term **or** long term outcome • major known confounders	• identifier • admin info • condition • intervention • short term outcome • major known confounders • long term outcome
**D. Completeness of data** **(percentage variables at** **least 95% complete). State** **when completeness was last** **determined:**	Few (<50%) or unknown	Some (50–79%)	Most (80–97%)	All or almost all (>97%)
**E. Form in which continuous** **data (excluding dates)** **are collected (percentage** **collected as raw data)**	Few (<70%) or unknown	Some (70–89%)	Most (90–97%)	All or almost all (>97%) or no continuous data collected
**F. Use of explicit definitions** **for variables**	None	Some (<50%)	Most (50–97%)	All or almost all (>97%)
**G. Use of explicit rules for** **deciding how variables are** recorded*	None	Some (<50%)	Most (50–97%)	All or almost all (>97%)
**H. Reliability of coding of** **conditions and interventions.** **State when and how it was** **most recently tested:**	Not tested	Poor	Fair	Good
**I. Independence of** **observations of primary** **outcome**	Outcome not included or independence unknown	Observer neither independent nor blinded to intervention	Independent observer not blinded to intervention	Independent observer blinded to intervention or not necessary as objective outcome (e.g. death or lab test)
**J. Extent to which data are** **validated. State when and** **how it was last determined:**	No validation	Range or consistency checks	Range and consistency checks	Range and consistency checks plus external validation using alternative source

**Table 2.** Framework of procedures for the assurance of data quality in medical registries according to Arts *et al*. (2002).

CENTRAL COORDINATING CENTER	Score y/n	LOCAL SITES	Score y/n
**Prevention during set up and organization of registry**			
*At the onset of registry*		*At the onset of participating in the registry*	
Compose minimum set of necessary data items	yes	Assign a contact person	yes
Define data and data characteristics in data dictionary	yes	Check developed software for data entry and for extraction	yes
Draft a data collection protocol	yes	Check reliability and completion of extraction sources	yes
Define pitfalls in data collection	yes	Standardize correction of data items	yes
Compose data checks	yes	*Continuously*	
Create user-friendly case record forms	yes	Train (new) data collectors	yes
Create quality assurance plan	yes	Motivate data collectors	yes
*In case of new participating sites*	yes	Make data definitions available	yes
Perform site visit	yes	Place data and initials on completed forms	yes
Train new participants	yes	Keep completed case record forms	yes
*Continuously*		Data collection close to the source and as soon as possible	yes
Motivate participants	yes	Use the registry data for local purposes	yes
Communicate with local sites	yes	*In case of changes*	
*In case of changes (e.g. in data set)*		Adjust forms, software, data dictionary, protocol, etc.	yes
Adjust forms, software, data dictionary, protocol, training material, etc.	yes	Communicate with data collectors	yes
Communicate with local sites	yes		
			
**Detection during data collection**		*Continuously*	
*During import of data into central database*		Visually inspect completed forms	yes
Perform automatic data checks	yes	Perform automatic data checks	yes
*Periodically and in case of new participants*		Check completeness of registration	yes
Perform site visits for data quality audit (registry data-source data) and review local data collection procedures	yes		
*Periodically*			
Check interobserver and intraobserver variability	no		
Perform analyses of the data	yes		
			
**Actions for quality improvement**		*After receiving quality reports*	
*After data import and data checks*		Check detected errors	yes
Provide local sites with data quality reports	yes	Correct inaccurate data and fill in incomplete data	yes *
Control local correction of data errors	yes	Resolve causes of data errors	yes
*After data audit or variability test*		After receiving feedback	yes
Give feedback of results and recommendations	yes	Implement recommended changes	yes
Resolve causes of data error	yes	Communicate with personnel	yes

*Procedure may vary between individual registries

### Performance review

Features and functions of the platform pertaining to data capture, quality and management were described and made available to a total of six reviewers. To maximize insight into the registry network while minimize potential sources of bias, a variety of scorers were identified. Three reviewers were independent reviewers with established track records in high quality critical care registry implementation and research in both high-income settings and LMICs. Three scorers were members of the CCA coordinating team (LP, TR, AB). Independent reviewers had full access to documentation, reports, training material and platform code, pertinent to the quality assurance features of the registry. Scores of individual reviewers were averaged to derive the aggregated score. Census data was summarized as median and interquartile range, with summary tables for individual registry completeness performed using software Python (version 3.7) ^18^.

All encounters of care reported through the seven registries during a prespecified period of six months ( **June-December 2020**) were included. The selection of this time period enabled evaluation of established collaborating registries (Indian Registry of IntenSive care [IRIS] ^19^, Pakistan registry of intensive care [PRICE] ^20^ and Nepal Intensive Care Registry Foundation [NICRF]) ^6^, and the inclusion of newly implemented registries (Afghanistan, Bangladesh, Malaysia and Vietnam). Basic information on these registries is detailed in Table 3.

**Table 3.** Characteristics of clinical registries involved in the Critical Care Asia (CCA) network.

	All registries	Afghani stan ^#^	Bangladesh	India	Malaysia	Nepal	Pakistan	Vietnam
**Patient episodes**	**20,507**	553	392	4,675	465	2,951	10,972	1,237
**Number of ICUs**	**97**	6	2	18	3	8	55	5
**Number of beds**	**1169**	60	20	213	26	138	557	155
**Type of ICUs**								
Mixed ICU	**33**	5		13	2	6	7	5
MICU	**19**	1	1	1	1	1	12	
SICU	**20**						19	
CT ICU	**1**						1	
SARI ICU	**15**			1		1	13	
HDU	**2**		1				1	
Other	**7**			3			2	
**Completeness of recruitment %**	**100**	NA	100	95	100	100	100	100
** Units assessing completeness, %**	**77**	0	100	48	100	100	95	40
**Long term outcomes included**	**--**	no	no	yes *	no	no	yes *	no

Data is presented as median (IQR) or n(%). ^#^Data collection for Afghanistan started on 2020-07-02. The remaining registries had 6 months complete collection. Abbreviations: ICU, intensive care unit; MICU, medical ICU; SICU, surgical ICU; CT ICU, cardio-thoracic ICU; SARI, severe acute respiratory infection; HDU, high dependency unit *Live in some participating ICUs

### Registry structure overview

**Figure 1.**
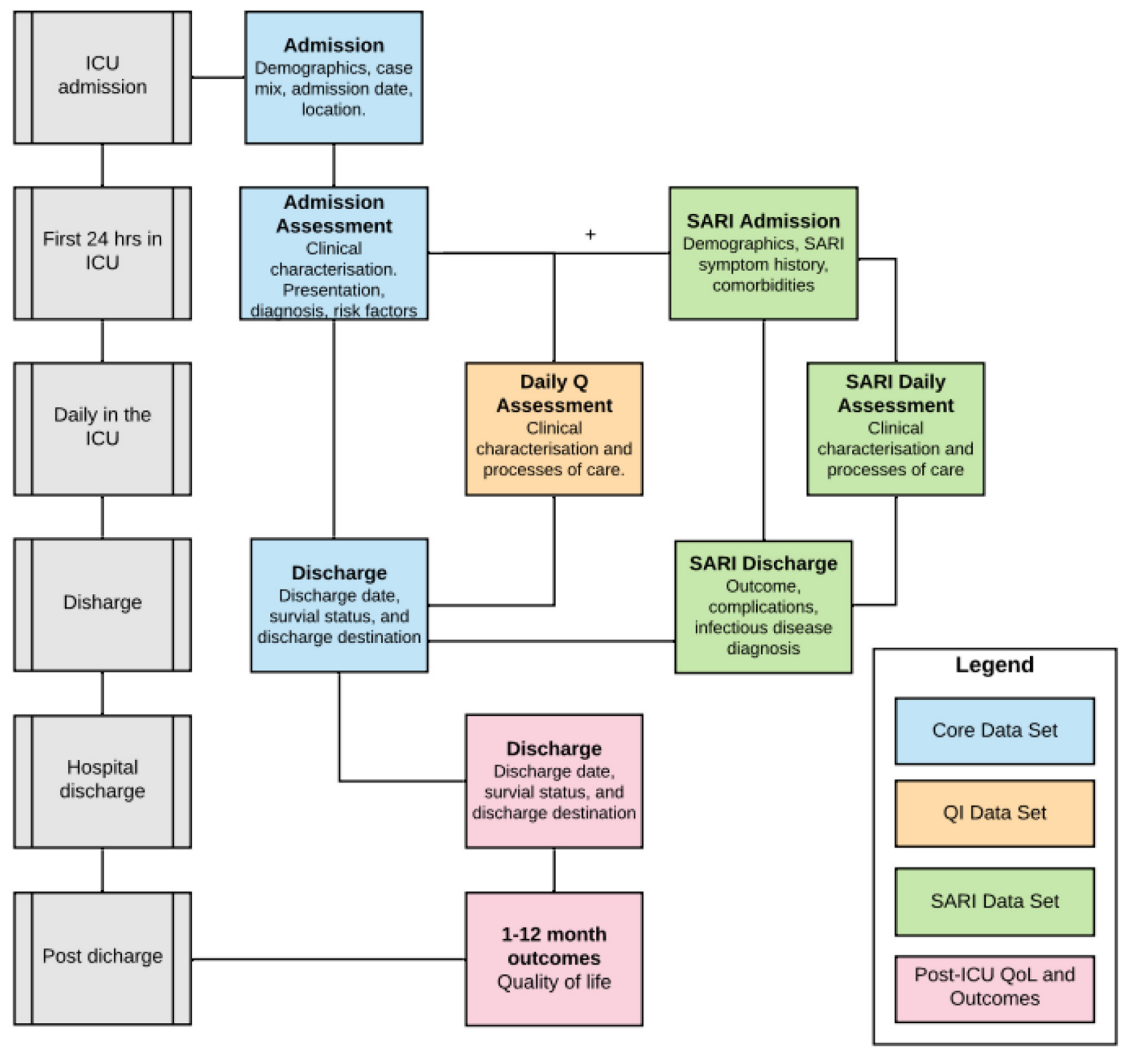
CRIT CARE ASIA registries modular data structure. Abbreviations: Q, quality; QI, quality improvement; ICU, intensive care unit (or any hospital unit involved in the project); SARI, severe acute respiratory infection; QoL, quality of life. Only the CORE data set is standard for all sites, while other data modules are optional.

Registry structure for established registries in India, Pakistan and Nepal was already published ^15, 19, 21^. In brief, the CCA platform has a modular structure, where a core dataset of 33 variables captured within the first 24 hours of admission to ICU and 5 variables at discharge, provides episodic information to enable evaluation of case mix, acuity, organ support and outcomes ^19, 22^. Additional modules complement the core data set providing stakeholders with a mechanism for embedding measures to evaluate care processes synonymous with care quality, and undertake observational and interventional research ( Figure 1). The registry platform has a customisable user mobile and desktop interface and accessible data entry support tools. Minimum data connectivity requirements (3G data and offline function) along with downloadable data exports facilitate the registries adoption in settings which may previously have failed to implement digital systems due to poor internet coverage or limited access to hardware. Integrated analytics dashboards and reports displaying trends in information, activity and quality indicators provide a mechanism for service reporting and cycles of audit and feedback with the clinical teams ^15^.

The network has a federated system for registry data storage, whereby national registries house their data and are supported to establish infrastructure and skills to manage and curate data. All anonymised registry and trial data is backed up to a central server. A summary of registry implementation procedures reported using the template for intervention description and replication (TIDieR) checklist is detailed in the extended data ^18^.

### Data collection procedures

Data is recorded prospectively and extracted directly from patient charts by data collectors daily and contemporaneous to clinical care. Laboratory tests are reported in the ICU’s routine unit of measurement and harmonised to a single measure. A comprehensive field specification and data collection guide are made available to all stakeholders through the platform. Data collectors are remotely trained prior to commencing data collection using a demo platform and ongoing 24 hr online support is available. Follow up meetings are offered weekly to enable ongoing feedback and improvement regarding data quality and support with registry led research and audit. Census checks with independent admission data are used to monitor cohort inclusion daily or weekly at users’ preference. The platform's existing internal data quality mechanisms, field completeness, value range validity and branching logic prompt users to missed or potentially spurious responses.

## Results

### Assessment of performance using the DoCDat criteria

A summary of the performance of the registries using the DoCDat criteria is shown in Table 4 ^18^, and compared to the average evaluation of other existing DoCDat databases ^11, 16^. The median score achieved by the registries across all criteria was 3.4 (minimum 1.4, maximum 4). Detailed scoring of each criterion is described below, while the score assigned by each external and internal reviewer is detailed in Table 5. An earlier version of this article can be found on medRxiv ( https://doi.org/10.1101/2021.07.10.21260243)

**Table 4.** Assessment of the Crit Care Asia (CCA) network registries according to the Directory of Clinical Databases (DoCDat) criteria.

	Crit Care Asia registries score ^#^	DoCDat database *
A. Representativeness of country	1.5 (1-2)	3 (2-4)
B.Completeness of recruitment	2.7 (2-3)	3 (1-4)
C. Variables included	3.3 (3-4)	3 (2-4)
D. Completeness of data	3.8 (3-4)	2 (1-3)
E. Collection of raw data	3.8 (3-4)	4 (4-4)
F. Explicit definitions	4 (4-4)	2 (1-4)
G. Explicit rules	3.8 (3-4)	3 (1-4)
H. Reliability of coding	3.7 (2-4)	1 (1-4)
I. Independence of observations	3.8 (3-4)	4 (2-4)
J. Data validation	3.5 (3-4)	3 (3-4)

# Average score of 6 independent reviewers, displayed as average (minimum and maximum scores attributed by individual scorers). *Extracted from reference ^11^

**Table 5.** Scoring overview of the external and internal reviewers according to the DoCDat criteria.

	External reviewer 1	External reviewer 2	External reviewer 3	Internal reviewer 1	Internal reviewer 2	Internal reviewer 3	Mean
A. Representativeness of country	1	2	2	2	1	1	1.5
B. Completeness of recruitment	3	2	3	3	2	3	2.7
C. Variables included	3	3	3	3	4	4	3.3
D. Completeness of data	4	4	4	3	4	4	3.8
E. Collection of raw data	4	3	4	4	4	4	3.8
F. Explicit definitions	4	4	4	4	4	4	4.0
G. Explicit rules	4	4	4	4	3	4	3.8
H. Reliability of coding	4	4	2	4	4	4	3.7
I. Independence of observations	4	4	4	4	4	3	3.8
J. Data validation	4	4	4	3	3	3	3.5
Overall mean							**3.4**

***A. Representativeness of country***. Mean score 1.5. Despite the high number of ICUs in several countries, the geographic spread inside each country was limited for all registries.

***B. Completeness of recruitment***. Mean score 2.7. Recruitment completeness i.e. the proportion of patients reported in the registry over the number of patients admitted to the ICU was >95% in all participating ICUs ( Table 3). Registry team members contact each ICU on a daily or weekly basis as preferred by the registry and validate admission, discharge and bed occupancy. The recruitment completeness was assessed through a dedicated section of the online platform. The process of daily or weekly validation of recruitment completeness was conducted in all but one registry, and in 77% of all ICUs ( Table 6).

**Table 6.** Completeness of recruitment by individual registry and month.

Month ^$^	Eligible censuses	Actually completed censuses	% of completed censuses	% of censuses with number of reported>admitted patients	Completeness of recruitment, median **	Completeness of recruitment, IQR_25	Completeness of recruitment, IQR_75
**BANGLADESH**							
06/2020	5	5	100	0	83	0	100
07/2020	8	6	75	17	100	85	100
08/2020	10	10	100	20	100	100	100
09/2020	8	8	100	25	100	100	101
10/2020	8	8	100	13	100	100	100
11/2020	10	10	100	0	100	94	100
12/2020	6	6	100	0	100	100	100
INDIA (IRIS)							
06/2020	46	35	76	28	100	79	104
07/2020	40	20	50	25	94	81	101
08/2020	50	20	40	25	100	82	102
09/2020	43	23	53	21	100	87	100
10/2020	44	17	39	41	100	95	109
11/2020	55	23	42	39	100	85	108
12/2020	33	16	49	31	85	60	105
MALAYSIA							
09/2020	3	3	100	33	100	100	150
10/2020	71	70	98	19	100	100	100
11/2020	90	87	97	6	100	100	100
12/2020	93	84	90	11	100	100	100
NEPAL (NICRF)							
06/2020	92	92	100	26.1	100	79	117
07/2020	124	122	98	21	100	100	100
08/2020	124	124	100	20	100	100	100
09/2020	120	120	100	18	100	100	100
10/2020	124	124	100	31	100	100	127
11/2020	148	145	98	19	100	100	100
12/2020	248	248	100	16	100	100	100
PAKISTAN (PRICE)							
09/2020	48	48	100	0	100	100	100
10/2020	241	235	97.5	0.4	100	100	100
11/2020	305	302	99	0.3	100	100	100
12/2020	183	183	100	0	100	100	100
VIETNAM							
11/2020	3	2	66	50	150	125	175

The “census” is the *weekly* comparison of the number of patients admitted to the ICU in a week against the number of patients entered in the registry. No units were collecting census during the study period in the Afghanistan registry. ^$^Only the months for which a census was reported are visualized in the table ** Number of recruited patients was calculated as number of reported patients divided by the number of admitted patients as identified by the census. Abbreviations: IRIS, Indian Registry of IntenSive care; PRICE, Pakistan registry of intensive care; NICRF, Nepal Intensive Care Registry Foundation

***C. Variables included***. Mean score 3.3. All seven registries reported the core data set and were able to derive severity of illness and prediction of mortality using published scores (Acute Physiology and Chronic Health Evaluation [APACHE] II and Tropical Intensive Care Score [TropICS]) ^23^. Variables included standardised diagnosis and comorbidities (Systematized nomenclature in Medicine - clinical terms [SNOMED CT] and Charlson comorbidity index), and outcomes at ICU and hospital discharge ( Table 7). Two registries (IRIS in India and PRICE in Pakistan) also collected medium to long term patient centred outcomes (i.e. after hospital discharge) and quality of life indicators such as the Euro quality of Life 5-dimensions tool (EQ5D-3L) ^24^ and scales for post traumatic stress disorders (PTSD).

**Table 7.** Completeness of data - core variables.

Form		Variable	Availability (%)
**Admission**	1	Patient name	100
	2	Medical record number	100
	3	Age	100
	4	Gender	100
	5	Date of admission to hospital	100
	6	Time of admission to hospital	100
	7	Date of admission to ICU	100
	8	Time of admission to ICU	100
	9	Readmission to ICU	100
	10	Admission type (operative vs. non operative)	100
	11	Admission diagnosis	100
	12	Comorbidities	100
	13	Confirmed or suspected SARI	99.4
**Admission assessment**	14	Ventilatory support (mechanical vs self ventilation)	97.6
	15	Route of ventilatory support (ETT vs tracheostomy vs NIV)	100
	16	Cardiovascular support	97.6
	17	Type and dose of vasoactive drug	96.6
	18	Use of sedatives	97.6
	19	Use of antibiotics	97.6
	20	Class of antibiotic	100
	21	Systolic blood pressure	97.6
	22	Diastolic blood pressure	97.6
	23	Respiratory rate	97.6
	24	Heart rate	97.6
	25	Body temperature	97.6
	26	Renal replacement therapy	97.5
	27	Glasgow coma scale	97.5
**Discharge**	28	Date of discharge	100
	29	Time of discharge	100
	30	Discharge status	100
	31	Discharge destination	100
	32	Cardiopulmonary resuscitation during stay	100
	33	Withdrawal of treatment	100

Abbreviations: ICU, intensive care unit; SARI, severe acute respiratory infection; ETT, endotracheal tube; NIV, non invasive ventilation.

***D. Completeness of variables***. Median score 3.8. All core variables were reported in the seven registries with < 5 % missingness, sustained over the 6-month period ( Table 7). Overall, the availability of the core data set was 98.9%. All vital signs had a completeness >97%, while the variable with lowest score regarded type and dose of vasoactive drugs (96.6%). 

***E. Capture of raw variables***. Median score 3.8. Raw data accounts for all fields in the core data set. Weekly meetings and 24/7 remote support between the CCA platform team and collaborating registries were reported using an online project management tool, which provided an audit trail of user queries, responses and platform development in response to recurring themes from user feedback.

***F. and G. Explicit rules for how variables are recorded***. Median scores 4.0 and 3.8 respectively. A detailed data dictionary complete with field specifications was available for all variables in the dataset and was uniform across the registries.

***H. Reliability of coding***. Mean score 3.7. The CCA platform’s use of SNOMED CT ( www.snomed.org) and Observational Medical Outcomes Partnership (OMOP) common data model mapping ( www.ohdsi.org), ensures international standardized nomenclature covering both diagnostic conditions and operative procedures in all collaborating registries. However, no intra-rater or inter-rater reliability testing was performed.

***I. Independence of observations***. Median score 3.8. The primary outcome assessment for all episodes of care, was observed independent of patient care and independent from the clinical team. Similarly, secondary outcomes pertaining to vital status as 30 days- up to one year following ICU admission were captured by investigators blinded to existing encounter data.

***J. Data validation***. Mean score 3.5. Data is validated internally according to the CCA dataset definitions. Fields are validated for completeness, consistency of response across sibling or parent-child fields. Inbuilt mandatory rules developed based on cycles of testing and analysis in CCA network sites ensure completeness of core dataset, and alerts within the user interface prompt users to complete supplemental fields. Illogicalities and inconsistencies in relational fields are minimised using inbuilt branching logic. Data validation reports, updated every 24hrs, are accessible to end users via the platforms reports interface. Clinicians and administrators can also interrogate the CCA data set directly by downloading reports, viewing data via the real-time dashboards, or by submitting requests for analyses to the CCA registry implementation team. Free text fields are used only to supplement predetermined menus which have been generated from pre-existing guidance e.g. for Center of Disease Control definitions, or for the Acute Physiology and Chronic Evaluation (APACHE) IV diagnostic codes).

### Assessment using the framework of procedures proposed by Arts *et al.* ^17^

The CCA platform fulfilled all criteria proposed by this framework, with the exception of 1 item ( Table 2) pertaining to the central coordinating center checking on interobserver variability. The scoring for this framework was homogeneous across all reviewers.

## Discussion

This independent evaluation of federated critical care registries from seven LMICs in Asia performed better than previously reported evaluations of multi centre databases using the DoCDat criteria ^2, 11^. Key components of the platform were standardised field specification, inbuilt validation at data entry, audit reporting on completeness, consistency and validity checks of the data. The greatest limitation of the registries when evaluated against the criteria were in national geographical coverage and the absence of source verification of data.

The representativeness criteria was the lowest scoring as the CCA network spread is inhomogeneous with large differences across countries. The primary goal of capturing outcomes information is to identify high-performance hospitals or health-care delivery systems in order to uncover the best practices responsible for their superior outcomes and seek to implement them in other settings. A limited coverage across the collaborating registries limits the ability to benchmark care nationally and internationally, but such benchmarking may have limited utility in healthcare systems in developing countries. This is due to both difficulty in capturing outcomes after ICU discharge and infeasibility of complex risk adjusted stratification. Although historically national coverage has been considered a key criterion to enhance data quality, we do not consider this to be the case for a federated network system spanning across several countries. The focus is on the community of practice rather than the extent of coverage, on the actual use of the data for unit level or multicenter quality improvement initiatives, audit and feedback rounds and clinical trials. Yet, efforts to increase expansion inside individual countries continue, with new centers joining the registry on a regular basis.

Some of the challenges faced by the CCA registry are specific to LMICS, others are more common and observed across registries worldwide ^11, 12^. Completeness of recruitment is still not assessed in one third of the CCA ICUs and limits the exact knowledge of patients missed by the registry. On the other hand, the patient census often was higher than the reported admitted patients on ICU admission books, questioning the reliability of routinary admission books as a representation of the exact count of admitted patients. Staffing and retention of dedicated data collectors are also recognized challenges faced by registries worldwide ^11, 12^.

Data collection, data entry and verification are frequently carried out by staff from diverse clinical or non-clinical backgrounds with verification of data accuracy that may be seldom performed at unit level. Despite no formal audit of a sample of medical records was performed, similar rates of discrepancies (i.e. around 5%) found in previous registries ^11, 25^. may be expected from the CCA federated registry system. Source data verification (SDV), whilst not a formal part of routine registry data quality assessment, is conducted on registry data, used for clinical trials. A powerful infrastructure for enabling clinical research in settings where trial resource and experience is limited, CCA collaborating centers participate in international clinical trials, including REMAP- CAP and MegaROX in part because the trial CRFs have been embedded into the registry platform. Up to 100% of study data for trial enrolled patients is subject to SDV. The operationalisation of clinical research through the registry platform is an important mechanism for assessing and improving overall data quality, and for establishing a culture of clinical audit, feedback and research whereby there is direct linkage of data collected to evaluating patient outcomes and delivering service improvement. What remains perhaps more uncertain is the reliability of the underlying source documentation. Reviewing source documentation (SDR) to assess the underlying quality of the data is largely absent from healthcare data internationally. Assessing documentation for patterns of data and deviations is likely to reflect significant biases in both what and when information (individual data and clinical events) is recorded as clinical practice varies widely both within a given setting and internationally. Limitations and potential flaws in reliability of registry data have been highlighted in the past ^26^. Rigorous and regular assessments of registry data such as the one performed in this article may overcome some of these limitations. Continuous audit and analysis at unit, regional and national level also contribute to strengthening data collection and interpretation procedures.

With the increased use of registries for registry-embedded clinical trials and observational research there is a drive for improved data quality ^27^. In addition to the mandatory field completeness, range checks, primitive and entity data-type constraints, additional mechanisms are in place for data quality assurance: data version management, access control for curated data sets, role-based access, verified audit trails and source verification of data. Registries can also allow a better understanding of how close standard care arms are to routine care, through the validation of trial data in the context of pre-existing registry data. Finally, data interoperability across multinational registries is currently being facilitated by the increasing integration of international coding systems (e.g. SNOMED), use of Common Data Models and the participation in data sharing initiatives such as the Linking of Global Intensive Care (LOGIC) consortium ^28^.

The architecture of the CCA registry facilitates ICUs retaining ownership of submitted data. The CCA registry provides contributors with a platform for capture of unit level data using a common data structure, and enables real time analysis to inform clinical care and service delivery via dashboards and collated reports. In fact, ICU beds in Asian hospitals constitute an average 9% of hospital beds, highlighting the importance of reliable and comparable data ^29^. Leveraging the same data platform, ICUs can contribute patient and hospital de-identified data to the CCA for benchmarking, multi-centre research purposes and quality improvement. Investigator initiated research can also be started by ICU registry leads within the network and on approval and agreement of clinical and institutional collaborators.

Similarly to the DoCDat criteria, Arts *et al.* suggested the need for transparent data definitions, standardized data collection guidelines and central training of individuals involved in data collection ^17^. The CCA failed to meet one of the suggested criteria concerning the interobserver variability checks on collected data. This would require the data collection performed by different individuals with a subsequent check against the source files, a resource-intensive procedure that constitutes a challenge for all quality clinical registries ^27^. Yet, all the other domains pertaining to both the central coordinating center and peripheral ICUs were fulfilled. This provides factual endorsement for the federated system experimented by the CCA network of multiple registries with both national and international coordination.

Across the globe, registries are now being leveraged to support large scale multi-centre clinical trials and evaluate complex improvement interventions. Regarding trial recruitment, adapted registry platforms promote rapid onboarding, inform site selection and improve patient recruitment, and can facilitate study monitoring through inbuilt data quality and validation processes ^6^. Potential limitations of registry-based trials concern the controlling for confounding and bias ^30^. The CCA network is already supporting several of the REMAP-CAP arms trials ^6, 31, 32^, while also enabling observational and outcome research ^23^.

This study has some limitations. The assessment was limited to core data as this dataset was available throughout all registries in the network. While other data domains will presumably share similar infrastructure scoring, the completeness of data may vary. The assessment included registries with diverse size and experience, with aggregate scoring performed without emphasis on single registry’s scores and improvement points.

## Conclusions

The CCA federated registry system is a rapidly growing network that provides high quality ICU data concerning case mix, processes of care and clinical outcomes from seven Asian countries. The system had a high performance when assessed using rigorous predefined scoring systems tackling completeness, reliability, validity and organizational infrastructure. While representativeness and interobserver reliability checks were identified as potential areas for improvement, overall performance was equal to national registries in high income settings.

## Data availability

### Underlying data

Figshare: Registries performance CCAA NICSMORU 13-08-21. https://doi.org/10.6084/m9.figshare.15167406.v3 ^18^.

This project contains the following underlying data:

- Registries_performance_CCAA_NICSMORU_13_08_21_patient_data_entered_status.csv
- Registries_performance_CCAA_NICSMORU_13_08_21_census_data.csv
- Registries_performance_CCAA_NICSMORU_13_08_21_unit_infomation.csv
- CORE Data dictionary_CCAA.xlsx

For further information regarding the data and the CCA, please contact the CCA data access committee ( DAC@nicslk.com) and quote the manuscript, your institution and provide return correspondence information.

### Extended data

Fighsare: TIDieR checklist for ‘Performance evaluation of a multinational data platform for critical care in Asia’. https://doi.org/10.6084/m9.figshare.15167406.v3 ^18^.

Data are available under the terms of the Creative Commons Attribution 4.0 International license (CC-BY 4.0).

Archived analysis code as at time of publication: https://doi.org/10.6084/m9.figshare.15167406.v3 ^18^.

License: Creative Commons Attribution 4.0 International license (CC-BY 4.0).

## Collaborators

Collaborators are listed by registry in alphabetical order (Site leads are in **bold**):

### AFGHANISTAN:

Ahmad Seyar Quraishi, Meharnegar Haqyar Mohammadi, Ahmad Zekria Sherzai (Wazir Akbar Khan Hospital, Kabul), **Ghulam Rahim Awab**, Noorullah Ahmadzai, Guldad Khan Safi (Nangarhar Regional Hospital, Jalalabad), Mirwais Azizi (Ali Jenah Hospital, Kabul), Asilah Hedayat (Herat Regional Hospital, Herat); **Naseebullah Barekzai**, Dawood Safi (Indira Gandhi Children’s Hospital, Kabul).

### BANGLADESH:

**Aniruddha Ghose, Ranjan Kumar Nath,** Selim Kashem Chowdhury, Smriti Kona Debi, Kamrun Akter, Safiqul Mostafa Chy (Chattogram Medical College Hospital, Chattogram).

### INDIA (Indian Registry of IntenSive care -- IRIS):

**Devachandran Jayakumar**, Suresh Babu Masilamani (Apollo Speciality Hospital, Chennai); **Bharath Kumar,** Augustian James, Nagarajan Ramakrishnan, Ramesh Venkataraman, Lakshmi Ranganathan (Apollo Main Hospital, Chennai); **Meghena Mathew,** Revathi Kandeepan (Apollo First Med Hospital, Chennai); **Ebenezer Rabingrarajan,** Madhu Shree, Usha Rani Chandramohan (Apollo Speciality Hospital, Vanagaram); **Jaganathan Selvanayagam,** Thirumalai Sambath (Mehta Hospital, Chennai); **Mathew Pulicken,** Milan Mathew (Pushpagiri Medical College Hospital, Kerala); **Rakesh Lakshmappa**, Karthik Shivani Lokeshappa (Nanjappa Multispecialty Hospital, Karnataka); **Raymond Savio,** Sristi Patodia, Premnath Balakrishnan (Apollo Proton Cancer Center); **Kishore Mangal,** Disha Chandel (Eternal Hospital, Jaipur Rajasthan); **Deepak Vijayan,** Krishna Priya, (KIMS, Kerala); **Rajyabardhan Patnaik,** Kasi Chinni Krishna (ISPAT General Hospital, Rourkela); **Kavita Kamineni,** Saradha Chirravuri (ABC Hospital, Visakhapatnam); **Swagatha Tripathi,** Kasturi Sanyal (AIIMS, Bhuvaneshwar); **Zubair Mohamed**, Anna Paul (Amrita Institute of Medical Sciences).

### NEPAL (Nepal Intensive Care registry Foundation -- NICRF):

**Diptesh Aryal,** Sanjeet K Shrestha, Kishor Khanal, Ashim Regmi, Namrata Rai, Kanchan Koirala, Kaveri Thapa, Krisha Dheke, Manisha Maharjan (Nepal Mediciti Hospital, Lalitpur); **Subhash Acharya**, Kabita Sitoula, Asmita Pokhrel, Namrata Shrestha, Saraswoti Sharma, Bimala Make, Arati Phuyal, Radhika Maharjan, Sabi Bajracharya, Roshni Thapa, Binita Bhattarai (Tribhuvan University Teaching Hospital, Kathmandu); **Sabin Koirala, Hem Paneru,** Sujata Chauhan, Angela Lamichhane, Alina Lamichhane, Sangita G.C, Swastika Phuyal, Crystal Maharjan, Anusha Subedi, Bini Kayastha, Kabita Khadka, Rakshya Karki Pratibha, Paudel XX (Hospital for Advanced Medicine and Surgery HAMS, Kathmandu); **Sushil Khanal,** Samina Amatya, Pujan Rajbhandary, Bina Bhattarai, Sabita Shrestha Sharmila Mali XX (Grande International Hospital); **Basanta Gauli,** Nisha Bhandari, Babita Ghimire, Asmita Kaini (Chitwan Medical College, Chitwan); **Bipin Karki, Pramesh Shrestha,** Roshni Karki, Sabina Dhakal, Mandira Thapa, Sarita Tamang (Om Hospital, Kathmandu); **Shubha Kalyan Shrestha,** Roshni Kafle, Kalpana Gurung (Karuna Hospital, Kathmandu); **Sanjay Lakhey**, Anita Bashya, Prajina Malla, Jeeya Deuja (B&B Hospital, Lalitpur); **Anand Thakur,** Radhika Maharjan, Sachita Maharjan, Subina Maharjan Yashu (Nidan Hospital, Lalitpur); **Raju Shrestha,** Pratima Sigdel, Merina Pradhan (B & C Hospital, Lalitpur).

### MALAYSIA:

**Mohd Basri Mat-Nor,** Azrina Md Ralib, Nurhafizah Zarudin, Fatimah Mohamad (IIUMMC - International Islamic University Malaysia Medical Center, Kuala Lumpur); **Mohd Zulfakar Bin Maslan,**Wan Fadzlina Wan Ahmad Shukeri, Amir Asyraf (Hospital Universiti Sains Malaysia, Kota Bharu); **Noor Airini Ibrahim,**Noor Fazlina (Hospital Pengajar UPM, Pengajar); **Dr Mohd Shahnaz Hasan, Dr Nor'azim Mohd Yunos,** Dr Rafidah Atan, Yip Hing Wa (Universiti Malaya Medical Center, Kuala Lumpur).

### PAKISTAN (Pakistan Registry of Intensive Care -- PRICE):

**Ashok Kumar, Mukesh Kumar, Quratul Ain Khan, Osama Khalid, Ali Raza, Ali Abbas,** Akash Thakrani, Noor Hassan, Ilyas Shehzad, Samad Ali (Ziauddin Group of Hospitals, Karachi); **Dr Attaur Rehman,** Amir Khan, Ahmed Zia, Farhan Khan (Patel Hospital, Karachi); **Nawal Salahuddin, Amin Khawaja, Mohammad Imran, Sobia Masood,** Vinod Kumar (National Institute of Cardiovascular Diseases, Karachi); **Tanvir Alam,** Noor Hassan (Civil Hospital, Karachi); **Nadeem Muneer,** Vinod Kumar (Jinnah Post-Graduate Medical Center, Karachi); **Aneela Altaf Kidwai, Aftab Ahmed,** Amir Khan (Abbasi Shaheed Hospital, Karachi); **Rashid Nasim Khan,** Jhonsan Shahzad (Darul Sehat Hospital, Karachi); **Saeeda Haider, Junaid Patel, Fivzia Herekar,** Arther John, Yasir Rehman (The Indus Hospital, Karachi); **Fakhir Raza Haidri,** Fawadur Rehman, (SIUT, Karachi); **Muhammad** **Nasir Khoso,** Noor Hassan (South City Hospital, Karachi); **Saleh Khaskheli,** Muhammad Ibrahim (Peoples Medical University, Nawabshah); **Kashif Memon,** Mohsin Ali (Liaquat University Hospital, Hyderabad); **Maqsood Meher,** Afzal Ahmed (GMMM Teaching Hospital, Sukkur); **Sayed Muneeb Ali, Rana Imran Sikandar,** Imtiaz Ali Shah (Pakistan Institute of Medical Sciences, Islamabad) **; Liaquat Ali, Muhammad Ashraf Zia, Moazzam Tarar, Ahmed Ranjha,** Basit Ali, Shahryar Maqsood (Jinnah Hospital, Lahore); **Ahmed Farooq,** Shahbaz Ikram (Doctors Hospital, Lahore); **Arshad Taqi,** Junaid Anwar (National Hospital & Medical Center, Lahore); **Jodat Saleem, Irfan malik, Rehan Niazi,** Shahryar Maqsood (Lahore General Hospital, Lahore); **Naseem Ali Shah** (Hameed Latif Hospital, Lahore); **Kamran Cheema, Mazhar Ali Naqvi,** Basit Ali (Services Hospital, Lahore); **Iqbal Hussain, Mobin Chaudhary,** Muddasir Qadir (Pakistan Kidney and Liver Institute, Lahore); **Sairah Sadaf,** Anjum Saleem (Sheikh Zayed Medical College, Rahim Yar Khan) **; Muhammad Hayat, Arslan Rahatullah, Muhammad Kamran,** Farman Ali Khan, Imran Khan (North West General Hospital, Peshawar) **; Muhammad Sheharyar, Sajjad Orakzai,** Imran Khan, Zafar Iqbal Khatak (Lady Reading Hospital, Peshawar) **; Imran ul Haq,** Farman Ali Khan (Khyber Teaching Hospital, Peshawar).

### VIETNAM:

**Vu Dinh Phu, Dong Phu Khiem, Pham Van Phuc,** Doan Duy Thanh, Trinh Lan Huong, Dang Van Duong, Tran van Kien, Mac Duy Hung, Vo Duc Linh (National Hospital for Tropical Diseases, Hanoi); **Nguyen Thien Binh**, Nguyen Thi Thu Van, Vy Thi Thu Luan, Lien Thi Xuan Nga, Nguyen Thi Dieu Vy, Vuong My Dung, Cao Thi Lan Huong, Ho Ngoc Bao, Huynh Nhat Anh, Nguyen Hoang Huy, Truong Huynh Tan Phu, Nguyen Hong Ngoc, Tran Thi Kim Anh, Doan Minh Nhut (Trung Vuong Hospital, Ho Chi Minh); **Dinh Minh Duc, Vo Thi Dung,** Huynh Nguyet, Truong Dai Lan Ngoc, Nguyen Thuy Hang, Nguyen Tan Khanh (Dong Thap Hospital, Dong Thap); **Duong Bich Thuy,** (Hospital for Tropical Diseases, Ho Chi Minh City); **Yen Lam Minh,** Doan Bui Xuan Thy, Tran Minh Duc, Louise Thwaites, Nguyen Thanh Ngoc, Luu Phuoc An (Oxford University Clinical Research Unit).

### NICS MORU Crit Care Asia Network Registry Coordination Team:

Udara Attanayake, Sri Darshana, Kaumali Gimhani, Judy Ann Gitahi, Pramodya Ishani, Chamira Kodippily, Issrah Jawad, Shiekh Mohiuddin, Upule Pabasara, Dilanthi Priyadarshani, Disna Pujika, Aasiyah Rashan, Sumayyah Rashan, Thalha Rashan, Shoba Sathasivam, Timo Tolppa, Ishara Udayanga.

## References

[1] Kruk ME Gage AD Arsenault C : High-quality health systems in the Sustainable Development Goals era: time for a revolution. *Lancet Glob Health.* 2018;6(11):e1196–1252. 10.1016/S2214-109X(18)30386-3 30196093 PMC7734391

[2] Zampieri FG Soares M Borges LP : The Epimed Monitor ICU Database®: a cloud-based national registry for adult intensive care unit patients in Brazil. *Rev Bras Ter Intensiva.* 2017;29(4):418–426. 10.5935/0103-507X.20170062 29211187 PMC5764553

[3] Haniffa R Mukaka M Munasinghe SB : Simplified prognostic model for critically ill patients in resource limited settings in South Asia. *Crit Care.* 2017;21(1):250. 10.1186/s13054-017-1843-6 29041985 PMC5645891

[4] Soares M Bozza FA Angus DC : Organizational characteristics, outcomes, and resource use in 78 Brazilian intensive care units: the ORCHESTRA study. *Intensive Care Med.* 2015;41(12):2149–60. 10.1007/s00134-015-4076-7 26499477

[5] Pisani L Algera AG Neto AS : Epidemiological Characteristics, Ventilator Management, and Clinical Outcome in Patients Receiving Invasive Ventilation in Intensive Care Units from 10 Asian Middle-Income Countries (PRoVENT-iMiC): An International, Multicenter, Prospective Study. *Am J Trop Med Hyg.* 2021;104(3):1022–1033. 10.4269/ajtmh.20-1177 33432906 PMC7941813

[6] Aryal D Beane A Dondorp AM : Operationalisation of the Randomized Embedded Multifactorial Adaptive Platform for COVID-19 trials in a low and lower-middle income critical care learning health system. *Wellcome Open Res.* 2021;6:14. 10.12688/wellcomeopenres.16486.1 33604455 PMC7883321

[7] Black N : High-quality clinical databases: breaking down barriers. *Lancet.* 1999;353(9160):1205–6. 10.1016/S0140-6736(99)00108-7 10217078

[8] World Health Organization: WHO Global diffusion of eHealth: making universal health coverage achievable. World Health Organization Geneva.2016. Reference Source

[9] WHO Packages DHIS2. (accessed May 23, 2021). Reference Source

[10] Hung YW Hoxha K Irwin BR : Using routine health information data for research in low- and middle-income countries: a systematic review. *BMC Health Serv Res.* 2020;20(1):790. 10.1186/s12913-020-05660-1 32843033 PMC7446185

[11] Stow PJ Hart GK Higlett T : Development and implementation of a high-quality clinical database: the Australian and New Zealand Intensive Care Society Adult Patient Database. *J Crit Care.* 2006;21(2):133–41. 10.1016/j.jcrc.2005.11.010 16769456

[12] Harrison DA Brady AR Rowan K : Case mix, outcome and length of stay for admissions to adult, general critical care units in England, Wales and Northern Ireland: the Intensive Care National Audit & Research Centre Case Mix Programme Database. *Crit Care LondEngl.* 2004;8(2):R99–111. 10.1186/cc2834 15025784 PMC420043

[13] MEASURE Evaluation: Routine Health Information Systems: A Curriculum on Basic Concepts and Practice - Syllabus - MEASURE Evaluation. (accessed May 10, 2021). Reference Source

[14] CRIT CARE ASIA: Establishing a critical care network in Asia to improve care for critically ill patients in low- and middle-income countries. *Crit Care LondEngl.* 2020;24:608. 10.1186/s13054-020-03321-7 PMC7558669 33059761

[15] Beane A Silva APD Athapattu PL : Addressing the information deficit in global health: lessons from a digital acute care platform in Sri Lanka. *BMJ Glob Health.* 2019;4(1):e001134. 10.1136/bmjgh-2018-001134 30775004 PMC6352842

[16] Black N Payne M : Directory of clinical databases: improving and promoting their use. *Qual Saf Health Care.* 2003;12(5):348–52. 10.1136/qhc.12.5.348 14532366 PMC1743755

[17] Arts DGT Keizer NFD Scheffer GJ : Defining and improving data quality in medical registries: a literature review, case study, and generic framework. *J Am Med Inform Assoc.* 2002;9(6):600–11. 10.1197/jamia.m1087 12386111 PMC349377

[18] Beane A : Registries performance CCAA NICSMORU 13-08-21. *figshare.* Dataset.2021. 10.6084/m9.figshare.15167406.v3

[19] Adhikari NKJ Arali R Attanayake U : Implementing an intensive care registry in India: preliminary results of the case-mix program and an opportunity for quality improvement and research. *Wellcome Open Res.* 2020;5:182. 10.12688/wellcomeopenres.16152.2 33195819 PMC7642994

[20] Hashmi M Beane A Taqi A : Pakistan Registry of Intensive CarE (PRICE): Expanding a lower middle-income, clinician-designed critical care registry in South Asia. *J Intensive Care Soc.* 2019;20(3):190–195. 10.1177/1751143718814126 31447910 PMC6693123

[21] CRIT Care Asia: Hashmi M Beane A : Leveraging a Cloud-Based Critical Care Registry for COVID-19 Pandemic Surveillance and Research in Low- and Middle-Income Countries. *JMIR Public Health Surveill.* 2020;6(4):e21939. 10.2196/21939 33147162 PMC7717923

[22] Hashmi M Taqi A Memon MI : A national survey of critical care services in hospitals accredited for training in a lower-middle income country: Pakistan. *J Crit Care.* 2020;60:273–278. 10.1016/j.jcrc.2020.08.017 32942162 PMC7441021

[23] TirupakuzhiVijayaraghavan BK Priyadarshini D Rashan A : Validation of a simplified risk prediction model using a cloud based critical care registry in a lower-middle income country. *PLoS One.* 2020;15(12):e0244989. 10.1371/journal.pone.0244989 33382834 PMC7775074

[24] Rabin R Gudex C Selai C : From translation to version management: a history and review of methods for the cultural adaptation of the EuroQol five-dimensional questionnaire. *Value Health.* 2014;17(1):70–6. 10.1016/j.jval.2013.10.006 24438719

[25] Herbert MA Prince SL Williams JL : Are unaudited records from an outcomes registry database accurate? *Ann Thorac Surg.* 2004;77(6):1960–4; discussion.1964–1965. 10.1016/j.athoracsur.2003.12.018 15172246

[26] Perner A Bellomo R Møller MH : Is research from databases reliable? No. *Intensive Care Med.* 2019;45(1):115–117. 10.1007/s00134-018-5073-4 30649588

[27] Litton E Guidet B de Lange D : National registries: Lessons learnt from quality improvement initiatives in intensive care. *J Crit Care.* 2020;60:311–318. 10.1016/j.jcrc.2020.08.012 32977140

[28] Dongelmans DA Pilcher D Beane A : Linking of global intensive care (LOGIC): An international benchmarking in critical care initiative. *J Crit Care.* 2020;60:305–310. 10.1016/j.jcrc.2020.08.031 32979689

[29] Arabi YM Phua J Koh Y : Structure, Organization, and Delivery of Critical Care in Asian ICUs. *Crit Care Med.* 2016;44(10):e940–948. 10.1097/CCM.0000000000001854 27347762

[30] Frieden TR : Evidence for Health Decision Making - Beyond Randomized, Controlled Trials. *N Engl J Med.* 2017;377(5):465–475. 10.1056/NEJMra1614394 28767357

[31] Angus DC Derde L Al-Beidh F : Effect of Hydrocortisone on Mortality and Organ Support in Patients With Severe COVID-19: The REMAP-CAP COVID-19 Corticosteroid Domain Randomized Clinical Trial. *JAMA.* 2020;324(13):1317–1329. 10.1001/jama.2020.17022 32876697 PMC7489418

[32] REMAP-CAP Investigators; Gordon AC Mouncey PR : Interleukin-6 Receptor Antagonists in Critically Ill Patients with Covid-19. *N Engl J Med.* 2021;384(16):1491–1502. 10.1056/NEJMoa2100433 33631065 PMC7953461

