# Supplementary material for: Performance evaluation of a multinational data platform for critical care in Asia
Source: Wellcome Open Res. 2022 Jul 11;6:251. Originally published 2021 Oct 1. [Version 2] doi: 10.12688/wellcomeopenres.17122.2 (PMC8812332; doi:10.12688/wellcomeopenres.17122.2)
Supplement: Peer review history

# Peer review: Reviewer response for version 1

**DOI:** 10.21956/wellcomeopenres.18913.r46344

**Published:** 2022-02-02

**Authors:** Stefano Finazzi

**Affiliations:** 1 Clinical Data Science Laboratory, Department of Public Health, Mario Negri Institute of Pharmacological Research IRCCS, Ranica, Italy

The authors present a very important and interesting experience about a new platform for data collection of ICU patients in several countries. The quality of the data collection system is assessed by independent reviewers through a few scores. The project is really well-managed and the paper very well-written. All relevant issues were considered and limitations are properly discussed.

Is the work clearly and accurately presented and does it cite the current literature?

Yes

If applicable, is the statistical analysis and its interpretation appropriate?

Yes

Are all the source data underlying the results available to ensure full reproducibility?

No source data required

Is the study design appropriate and is the work technically sound?

Yes

Are the conclusions drawn adequately supported by the results?

Yes

Are sufficient details of methods and analysis provided to allow replication by others?

Yes

Reviewer Expertise:

Clinical data science

I confirm that I have read this submission and believe that I have an appropriate level of expertise to confirm that it is of an acceptable scientific standard.

# Peer review: Reviewer response for version 1

**DOI:** 10.21956/wellcomeopenres.18913.r47253

**Published:** 2021-11-29

**Authors:** Paul Young

**Affiliations:** 1 Medical Research Institute of New Zealand, Wellington, New Zealand

This study describes a performance evaluation of the Critical Care Asia Registry using a recognised framework. This technical report is well written and thorough. As outlined, the framework that has been used is well-establish. The greatest uncertainty is probably about whether the data that are entered into the registry are truly an accurate reflection of what is in the source data. This is a concern in all registries but could conceivably be a particularly troublesome issue in lower income countries where resources are more limited. My only significant comment is whether more attention to this point in the Discussion would be appropriate.

Is the work clearly and accurately presented and does it cite the current literature?

Yes

If applicable, is the statistical analysis and its interpretation appropriate?

Yes

Are all the source data underlying the results available to ensure full reproducibility?

Yes

Is the study design appropriate and is the work technically sound?

Yes

Are the conclusions drawn adequately supported by the results?

Yes

Are sufficient details of methods and analysis provided to allow replication by others?

Yes

Reviewer Expertise:

Intensive Care Medicine

I confirm that I have read this submission and believe that I have an appropriate level of expertise to confirm that it is of an acceptable scientific standard.
